# The different structure-function correlation as measured by OCT and octopus perimetry cluster analysis in intracranial tumor and glaucoma patients

**DOI:** 10.3389/fendo.2022.938952

**Published:** 2022-07-27

**Authors:** Xiaochun Li, Jiayin Qin, Xiaoguang Cao, Zeqin Ren, Ting Cui, Yongzhen Bao

**Affiliations:** ^1^Department of Ophthalmology, Peking University People’s Hospital, Eye Diseases and Optometry Institute, Beijing Key Laboratory of Diagnosis and Therapy of Retinal and Choroid Diseases, College of Optometry, Peking University Health Science Center, Beijing, China; ^2^Department of Ophthalmology, Peking University International Hospital, Beijing, China

**Keywords:** visual field, retinal nerve fiber layer, intracranial tumor, glaucoma, correlation

## Abstract

**Background:**

To explore the correlation between visual field (VF) defect values and retinal nerve fiber layer (RNFL) thickness for intracranial tumor and glaucoma patients.

**Methods:**

Retrospective analysis is performed for the intracranial and glaucoma patients, whose VF defect values were measured with Octopus perimeter cluster analysis, RNFL thickness, ganglion cell layer (GCL) thickness, and optic disk parameters measured with swept-source OCT. The differences between VF and RNFL (including the data of optic disc) are calculated. The correlation between VF defect values and RNFL and GCL thickness are explored.

**Results:**

In total 43 eyes of 29 patients with intracranial tumor and 31 eyes of 19 patients with glaucoma were enrolled. The thickness of RNFL not only for the whole (360°), but also for the four quadrants was thinner in the glaucoma group than those of the intracranial tumor group (p<0.05), and similar to the thickness of GCL without significance (p>). There is no significant difference in VF for those two groups except glaucoma having lower sLV (p<0.05). A stronger correlation for mean deviations (MD)s of VF ten clusters and RNFL thickness of OCT twelve sectors is found in the glaucoma patients, but few in the intracranial tumor patients. Logistic regression also shows the loss of RNFL or increasing of vertical CDR and cup volume tending to the diagnosis of glaucoma and the irregular VF damage is inclined to the diagnosis of intracranial tumor.

**Conclusions:**

Intracranial tumor has a weak correlation between the RNFL thickness and Octopus VF MD, compared with that of glaucoma. OCT and Octopus VF might provide more helpful information for the differential diagnosis of intracranial tumor and glaucoma.

## Background

It is well-known that intracranial tumors can cause visual field (VF) damage when affecting the visual pathway. Also due to the retrograde degeneration, the compression of intracranial tumors can lead to the damage of ganglion cell axons, following the thinning of the retinal nerve fiber layer (RNFL) (1). Similar manifestations and conditions are also found in glaucoma eyes. With the variability of RNFL and VF manifestations, intracranial tumors are sometimes misdiagnosed as other diseases, such as glaucoma (2). A study showed that 6.5% of patients diagnosed with normal-tension glaucoma had clinically relevant compression of the anterior visual pathway (3). However, for intracranial tumors, early diagnosis is important to improve the possibilities of treatment and to reduce mortality (4). We strive to find an indicator to distinguish diagnoses between intracranial tumor and glaucoma.

Octopus perimeter (OP) is one of the most commonly used perimeters for assessing glaucoma and many neurological disorders. Cluster analysis is a special visual field analysis program conducted by OP, and divides OP into 10 clusters according to the distribution of RNFL. The built-in program automatically calculates the arithmetic mean value of mean deviation (MD) in each cluster. The cluster analysis can identify regional visual field defects while there is a small increase in MD. Some studies had explored the relationship between VF and RNFL in intracranial tumor and glaucoma patients (5–7)_ENREF_5. However, few were conducted with Octopus perimeter cluster analysis (8).

Our study is trying to analyze the structure-function correlation between the intracranial tumor and glaucoma patients, which was based on the OP ten clusters analysis and Topcon OCT twelve sectors RNFL thickness. Correlation analysis was performed to associate these VF clusters MD with RNFL sectors thickness measurements, and we try to find the difference in that correlation in the intracranial tumor and glaucoma patients.

## Methods

### Recruitment of patients, and inclusion and exclusion criteria

This retrospective study is conducted in the Peking University International Hospital, Beijing, China. The study is approved by the local ethical review board in accordance with the Declaration of Helsinki, and all patients provided informed consent.

Nineteen patients with glaucoma, and 29 patients with intracranial tumor, October 2018 to December 2020, are retrospectively enrolled in this study. For glaucoma patients, the inclusion criteria are diagnosed with the primary open-angle or angle-closed glaucoma, and BCVA is better or equal to 20/200. And the exclusion criteria are the acute phase of angle-closure glaucoma, high myopia (<-6.0D), the previous intraocular surgery, or any ocular diseases, which could induce the change of VF or RNFL, such as ocular trauma, corneal degeneration and dystrophy, optic nerve disease, macular or retinal disease. For the patients with intracranial tumor, the inclusion criterion is diagnosed with a pre-operative intracranial tumor, and BCVA is better or equal to 20/200. And the exclusion criteria are high myopia, previous intraocular surgery, or any ocular diseases, which could induce the change of VF or RNFL, such as glaucoma, ocular trauma, corneal degeneration, and dystrophy, optic nerve disease, macular or retinal disease.

### Clinical observations

Clinical history and routine clinical examination were performed by slit-lamp microscopy, indirect ophthalmoscopy, uncorrected (UCVA), and best-corrected visual acuity (BCVA) logMAR were tested, besides intraocular pressure (IOP), VF and RNFL. IOP was measured by noncontact tonometer (Canon TX-10/TX-F, Tokyo, Japan), slit lamp examination (Topcon SL-1E, Tokyo, Japan), and fundus examination (90 Dioptre, Volk Optical, Mentor, OH) with an undilated pupil. All tests were performed in the outpatient eye clinic.

### VF measurement

**Figure 1:**
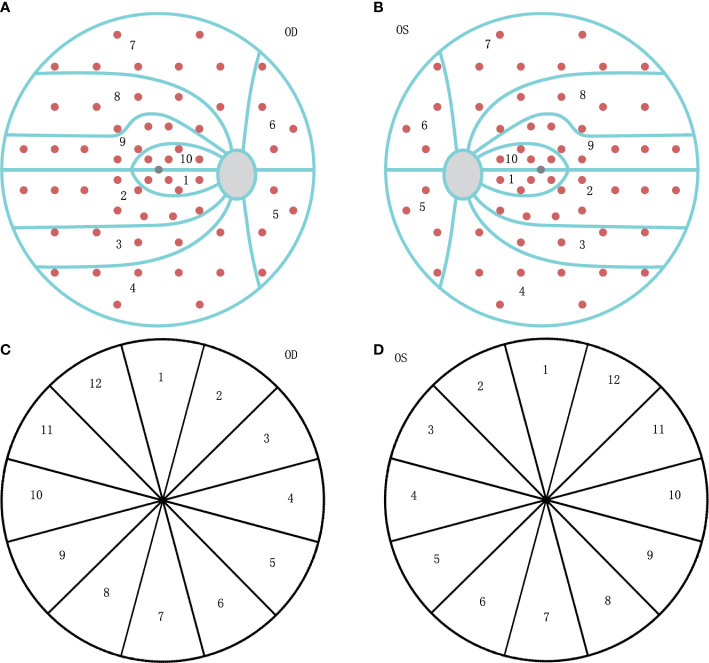
The diagram of visual field (VF) clusters and retinal nerve fiber layer (RNFL) clock-hour sector map. **(A)** The ten clusters are numbered 01 to 10 on the VF graph for the right eye (OD). **(B)** The ten clusters are numbered as 01 to 10 on the VF graph for the left eye (OS), mirrored as OD. **(C)** The clockwise number of twelve RNFL sectors on the OCT graph for the right eye (OD). **(D)** The counter-clockwise number of twelve RNFL sectors on the OCT graph for the left eye (OS), mirrored as OD.

VF was tested with Octopus 900 perimeter (Haag-Streit AG, formerly Interzeag AG, Schlieren, Switzerland). The program of White-on-White TOP strategy with 4/1000 asb III 100ms, and Octopus G Standard distribution of points was performed for those included patients. The acceptable criterion of a reproducible test is both a false-positive and false-negative response rate of less than 15%. The manufacturer-provided ten visual field clusters were used (**Figure 1**), and set as VF01-VF10, with calculated MD values. The clusters of the left eyes were mirrored and numbered as those of the right eyes, to ensure uniform handling of all data.

### RNFL measurement

Peripapillary RNFL thickness was measured at the same time without dilating the pupil, using DRI OCT Trion (Topcon Corp., Tokyo, Japan). RNFL measurements were categorized by clock hours, and labeled such that left eye sectors mirrored right eye sectors (**Figure 1**), set as Clock 1-12. Ganglion cell layer (GCL) thickness also could be read from the reports, and categorized as the rules of RNFL.

### Statistical analysis

The Kolmogorov–Smirnov test was used to verify the normality of data distribution. For quantitative comparisons between groups, we used the Student *t-test* for independent samples in parametric variables and the independent Mann-Whitney U test for the non-parametric variables. Pearson correlation coefficients were calculated to assess the relationship between variables. Binary logistic regression is calculated to assess the influence of VF and RNFL changes on the diagnosis of glaucoma or intracranial tumor. Statistical analyses were performed using SPSS statistical software for Windows (version 20.0, IBM-SPSS, Chicago, IL). The level of statistical significance was set at p<0.05.

## Results

Included patients are divided into two groups, intracranial tumor and glaucoma, due to their diagnosis. The intracranial tumor group contained 43 eyes of 29 patients (11 with pituitary adenoma, eight with meningioma, two with craniopharyngioma, three with glioma, one with ependymoma, one with metastatic tumor, one with cerebellar hemangioblastoma, one with cavernous hemangioma, and one with inflammatory pseudotumor). Of these 29 patients, at least 17 had tumors located near the optic chiasma, and at least 22 had tumors around and compressed the visual pathway directly. The tumor size of 21 patients was collected, with diameters from 12mm to 60mm. And the location of these intracranial tumors is the post-lateral geniculate body for 13 patients. The Glaucoma group contained 31 eyes of 19 patients (12 with primary open-angle glaucoma, and seven with primary angle-closed glaucoma). The mean ages of glaucoma and intracranial tumor groups are 56.21 ± 14.90 and 51.97 ± 14.11 years (p=0.326). The female ratios of glaucoma and intracranial tumor groups are no significant difference (9/10 and 18/11, p=0.315). IOP of the glaucoma eye was range 12.5 to 25.4 mmHg (17.50 ± 3.67mmHg), higher than that of the eyes with intracranial tumor was range 9.7 to 18.7 mmHg (13.79 ± 2.60mmHg) (p<0.01). All included patients, both intracranial tumor and glaucoma groups, had defects in their visual field. However, some of the included patients had suffered damage to RNFL thickness.

As the results of RNFL measured by TOPCON OCT in **Table 1**, the thickness of RNFL not only for the whole (360°) but also for the superior, inferior, nasal, and temporal quadrants are thinner in the glaucoma group than those of the intracranial tumor group (p<0.05). The thickness of temporal GCL is thinner in the glaucoma group than that of the intracranial tumor group without significance (p>0.05). The thickness of GCL for the whole and in the other quadrants of the glaucoma group has no significant difference from those of the intracranial tumor group (p>0.05). Moreover, the rim area, disc area, linear CDR, vertical CDR, and cup volume of optic disc measured by OCT all have significant differences for the two groups (p<0.05).

On the contrary, the results (global MD, MS) of VF measured with Octopus perimeter (**Table 1**),

**Table 1:** Data of VF and RNFL for the glaucoma and intracranial tumor groups.

Groups	Glaucoma	Intracranial tumor
N (eyes)	31	43
RNFL (360°) (μm)**	80.10±22.13	113.58±33.78
Superior quadrant of RNFL (μm)**	100.55±28.90	145.28±48.67
Inferior quadrant of RNFL (μm)**	92.32±39.34	150.12±43.98
Nasal quadrant of RNFL (μm)**	60.08±17.80	81.14±31.27
Temporal quadrant of RNFL (μm)*	66.65±19.63	78.21±23.16
GCL (360°) (μm)	39.00±6.58	39.27±5.28
Superior quadrant of GCL (μm)	36.85±8.96	37.08±7.40
Inferior quadrant of GCL (μm)	34.10±6.95	33.70±5.35
Nasal quadrant of GCL (μm)	39.46±9.48	38.10±6.23
Temporal quadrant of GCL (μm)	45.55±11.65	48.21±6.59
Rim area (μm^2^)**	0.67±0.41	1.59±0.90
Disc area (μm^2^) *	1.98±0.37	7.34±33.37
Linear CDR**	0.78±0.21	0.51±0.23
Vertical CDR**	0.79±0.22	0.49±0.23
Cup volume (μm^3^)**	0.41±0.29	0.12±0.14
MD of VF (dB)	17.77±7.40	16.89±5.40
MS of VF (dB)	10.02±7.12	10.97±5.35
sLV of VF (dB) *	4.80±2.29	6.15±2.56

Visual field (VF); Retinal nerve fiber layer (RNFL); Ganglion cell layer (GCL); Square root of loss variance (sLV); *p<0.05; **p<0.01.

**Table 2:** The correlation coefficient of VF and RNFL for the glaucoma group.

	Clock01	Clock02	Clock03	Clock04	Clock05	Clock06	Clock07	Clock08	Clock09	Clock10	Clock11	Clock12
VF01	-0.507**	-0.399*	-0.298	-0.045	-0.072	-0.368*	-0.335	-0.241	-0.550**	-0.403*	-0.520**	-0.392*
VF02	-0.537**	-0.521**	-0.367*	-0.122	-0.275	-0.516**	-0.434*	-0.337	-0.448*	-0.380*	-0.446*	-0.610**
VF03	-0.565**	-0.541**	-0.405*	-0.198	-0.271	-0.463**	-0.370*	-0.268	-0.331	-0.288	-0.301	-0.589**
VF04	-0.622**	-0.548**	-0.404*	-0.193	-0.185	-0.409*	-0.249	-0.196	-0.355*	-0.251	-0.232	-0.434*
VF05	-0.533**	-0.433*	-0.311	-0.135	-0.052	-0.215	-0.128	-0.172	-0.295	-0.177	-0.124	-0.225
VF06	-0.537**	-0.553**	-0.375*	-0.178	-0.163	-0.457**	-0.314	-0.346	-0.419*	-0.300	-0.320	-0.402*
VF07	-0.636**	-0.711**	-0.528**	-0.203	-0.316	-0.575**	-0.544**	-0.484**	-0.498**	-0.310	-0.366*	-0.463**
VF08	-0.644**	-0.670**	-0.477**	-0.136	-0.250	-0.551**	-0.596**	-0.559**	-0.632**	-0.398*	-0.408*	-0.494**
VF09	-0.600**	-0.639**	-0.406*	-0.113	-0.213	-0.530**	-0.643**	-0.647**	-0.692**	-0.461**	-0.463**	-0.500**
VF10	-0.513**	-0.524**	-0.117	0.042	-0.079	-0.403*	-0.549**	-0.530**	-0.754**	-0.517**	-0.439*	-0.426*

Visual field (VF); Retinal nerve fiber layer (RNFL); Square root of loss variance (sLV).

The clusters of VF and category of RNFL are set in **Figure 1** *p<0.05; **p<0.01.

**Table 3:** The correlation coefficient of VF and RNFL for the intracranial group.

	Clock01	Clock02	Clock03	Clock04	Clock05	Clock06	Clock07	Clock08	Clock09	Clock10	Clock11	Clock12
VF01	-0.128	-0.196	-0.099	0.001	0.103	0.025	0.326*	-0.190	-0.189	-0.211	-0.110	-0.096
VF02	-0.118	-0.117	-0.073	0.002	0.202	0.106	0.167	-0.048	-0.012	0.027	0.018	-0.071
VF03	-0.143	-0.201	-0.089	-0.043	0.154	0.059	0.161	0.017	0.043	0.040	0.038	-0.023
VF04	-0.110	-0.231	-0.079	0.000	0.189	0.072	0.270	-0.018	-0.071	-0.098	-0.056	-0.032
VF05	-0.206	-0.302*	-0.164	-0.081	0.163	-0.007	0.228	-0.207	-0.254	-0.215	-0.197	-0.169
VF06	-0.160	-0.258	-0.100	-0.051	0.029	-0.044	0.221	-0.217	-0.374*	-0.336*	-0.159	-0.110
VF07	-0.086	-0.225	-0.083	-0.081	0.109	-0.030	0.262	-0.090	-0.319*	-0.267	-0.143	-0.088
VF08	-0.007	-0.077	-0.064	0.012	0.205	0.051	0.380*	0.004	-0.184	-0.091	0.016	0.007
VF09	-0.091	-0.015	0.016	0.130	0.296	0.138	0.322*	-0.043	-0.072	0.023	0.039	-0.067
VF10	-0.187	-0.277	-0.149	-0.126	-0.070	-0.200	0.330*	-0.383*	-0.494**	-0.468**	-0.335*	-0.267

Visual field (VF); Retinal nerve fiber layer (RNFL); Square root of loss variance (sLV).

The clusters of VF and category of RNFL are set in **Figure 1** *p<0.05; **p<0.01.

**Table 4:** The influence of VF and RNFL on the diagnosis with the binary logistic regression.

	B	Wald	Sig.	Exp (B)OR	95% C.I. of EXP(B)/OR	
					lower	upper	
Thickness of RNFL	0.092	5.935	0.015	1.096	1.018	1.180	
Vertical CDR	-16.254	3.919	0.048	0.000	0.000	0.850	
Cup volume	-8.072	4.000	0.046	0.000	0.000	0.851	
sLV of VF	0.680	5.279	0.022	1.975	1.105	3.528	

Visual field (VF); Retinal nerve fiber layer (RNFL); squareroot loss variance (sLV); Ganglion cell layer (GCL); odds ratio (OR).

for the glaucoma group, have no statistically significant difference (p>0.05) from those of the intracranial tumor group. However, the sLV of VF for the glaucoma group is lower than that of the intracranial tumor group (p<0.05).

**Figure 2:**
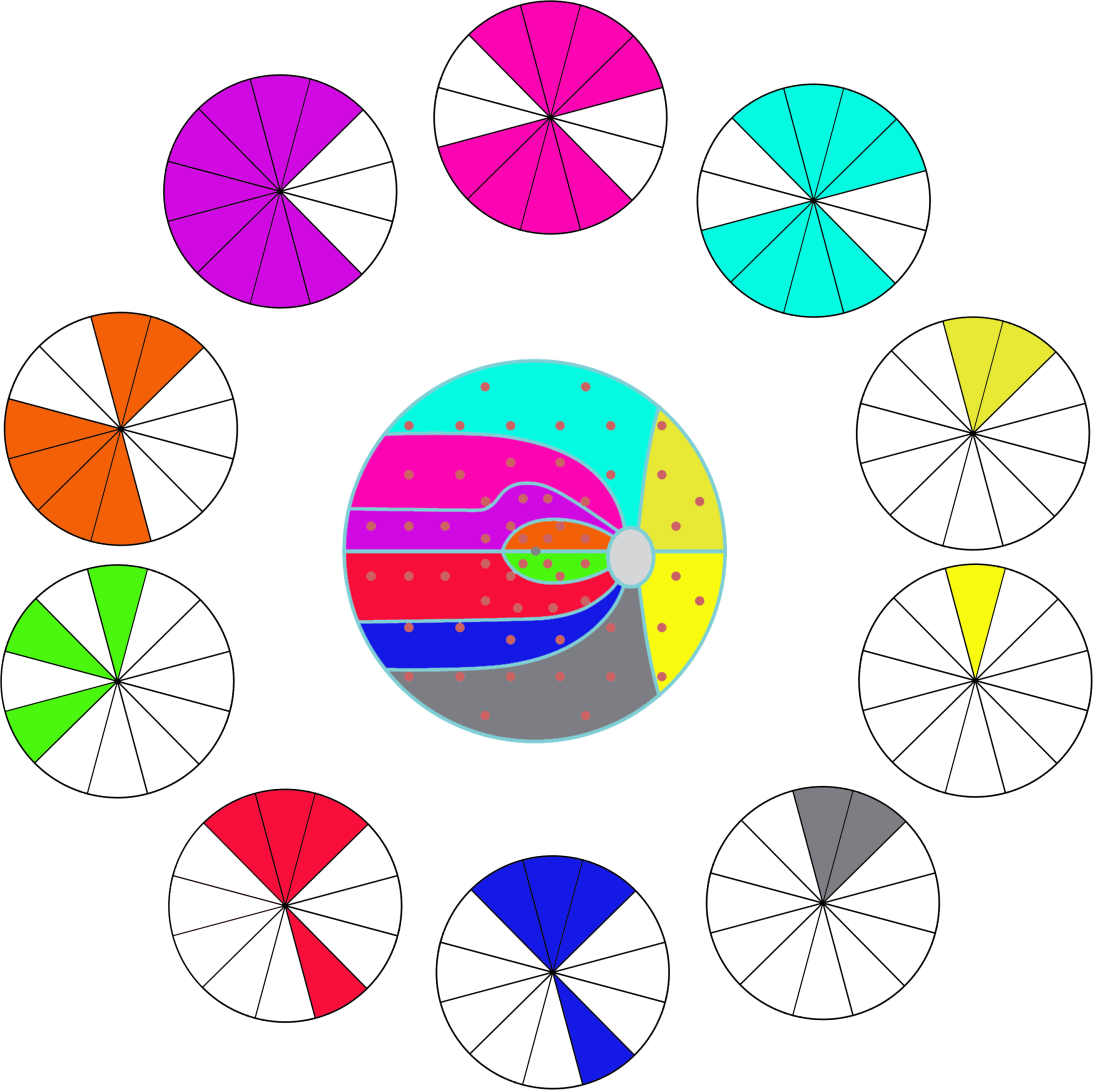
The correlation between visual field (VF) clusters and retinal nerve fiber layer (RNFL) clock-hour sectors. The same color indicates the correlation (coefficient value more than 0.45, Pearson’s Correlation Analysis) between VF clusters and RNFL sectors.

For the glaucoma group, the correlation coefficient between VF cluster MD and RNFL sector thickness is shown in **Table 2**, the clusters of VF and sectors of RNFL are set in **Figure 1**. And the strong correlations (value more than 0.45) could be observed in **Figure 2**. There were few correlations between the temporal VF of physiological blind spots and RNFL. The nasal VF of the physiological blind spot, that is, the binocular overlapping area of the human front, is significantly correlated with RNFL. As shown in **Table 3**, few correlations were observed between VF cluster MD and RNFL sector thickness for the intracranial tumor group, the figure of correlation is not drawn.

As the binary logistic regression, the thickness of RNFL and the damage of VF are included (**Table 4**). It shows the loss of RNFL tending to the diagnosis of glaucoma, which might be a weak factor as the value is around one. The vertical CDR and cup volume might be a stronger factor, with higher values tending to the diagnosis of glaucoma and lower tending to the diagnosis of intracranial tumor. And the irregular damage of VF is inclined to the diagnosis of intracranial tumor.

## Discussion

Visual field and OCT are the two most commonly used clinical examination methods for nerve injury disorders, such as glaucoma and intracranial tumor. The cluster analysis program in Octopus perimeter, which is designed according to the distribution of RNFL, can sensitively detect regional dysfunction when there are minimal visual field abnormalities. Perdicchi et al’s study showed within normal VF and abnormal ganglion cell complex (GCC) eyes of hypertension or early-stage glaucoma, all of the eyes showed abnormal results with cluster analysis (8). Some studies had shown the correlation between the structure and function in glaucoma patients with Humphrey or Octopus perimeter and OCT (9–11) _ENREF_9. Generally, these studies all found a topographic correlation in VF and OCT. But few studies have explored the topographic correlation in intracranial tumor patients with Octopus perimeter and OCT, fewer are in cluster analysis.

Our study shows a relative weak correlation between the MD of OP clusters with the thickness of RNFL in seven of ten OP clusters in those intracranial tumor patients (cluser1, 5, 6-10), most correlation coefficient absolute value is less than 0.45. While in the glaucoma patients, with each OP cluster, we find moderate correlations in more than one RNFL sector, which is similar to other studies (8, 11, 12) _ENREF_11. Also, the map (**Figure 2**) shows the topographic structure-function relationship in glaucoma. Previous studies also showed a moderate association between RNFL thickness in each sector with VF region either in Octopus or Humphrey perimetry in glaucoma patients (9–11), a structure-function map which is similar to ours was created. This result is highly consistent with the principle of human eye imaging. In the other words, the VF defect of a certain area can correspond to the thinning RNFL of the corresponding part. And the thinning of RNFL in a certain part can also correspond to VF damage in the corresponding area.

Our study shows a significantly lower vertical CDR and cup volume of the intracranial tumor group than those of the glaucoma group. This finding is quite consistent with the clinic feature of those two diseases. The visual field defect is due to the atrophy of intraocular nerve tissue in glaucoma eyes. The appearance of intraocular nerve tissue atrophy is the loss of rim area and an increase in the value of vertical CDR and cup volume. On the contrary, for intracranial tumor patients, the visual field defect usually is the result of central nervous system damage, and the appearance of the optic disc usually remains normal.

We assume the result may correlate with the different retinal ganglion cells (RGCs) damage mechanisms in glaucoma and intracranial tumor. It is well-known that glaucoma is characterized by the damage to RGCs axons initialing at the optic nerve head with different mechanisms, such as intraocular pressure mechanical compression, vascular disorders, immunologic influence, and oxidative stress. That may lead to direct retrograde damage to the RGCs, followed by the RNFL thinning and VF defect. Also, some studies show glaucoma optic disk change correlated with the intra-orbital optic nerve measurement and chiasmal size (13–15), which suggests glaucoma may also lead to anterograde degeneration post optic disk. Therefore, glaucoma may produce a bidirectional nerve injury from the optic nerve head.

While intracranial tumors cause retrograde degeneration on the visual pathway, the pathologic changes start from the distal axonal and progress centripetally, which is also found in other central nervous system pathologies such as cerebral infarction, head trauma, and multiple sclerosis (16–18). That includes two conditions. Tumor arising near the sella turcica causing the axonal or terminal lesions between the eye and lateral geniculate body leads to the direct retrograde degeneration (6) _ENREF_6. Whereas, tumors arising in the post lateral geniculate body cause damage to the optic radiation after the tertiary neurons in the visual pathway and lead to trans-synaptic retrograde retinal degeneration (TRD). It also causes the RNFL thinning and the optic nerve head vessel density to decrease (16, 19, 20). This is mechanically different from the damage to RGCs and axons in glaucoma.

Previous research showed the chiasmal lesion caused a more prominent optic nerve head vessel density decrease than the post-geniculate lesion, which indicated the direct retrograde degeneration might be more prominent than TRD (20). And our study shows the RNFL thinning is less prominent than glaucoma’ RGCs’ degeneration, both of the direct and trans-synaptic retrograde induced by the intracranial tumor. Whether the RNFL damage extent is negatively correlated with the distance from the initial site of injury to the RGCs remains unknown and needs more research to prove.

Another hypothesis is that the weak structure-function correlation in intracranial tumor patients is might due to the less injury of RNFL caused by direct or trans-synaptic retrograde. Our study yields two age and VF matched groups, of glaucoma and intracranial tumor, and shows more severe RNFL damage, smaller rim area, larger cup volume, and larger C/D (p<0.05) in the glaucoma patients. Also, some studies showed the optic chiasmal compression might cause the cell-inner plexiform layer to thin without RNFL changing in the early phase of some intracranial tumors (21, 22) _ENREF_21. However, Orman et al’s study showed, pituitary tumors might have RNFL thinning and RGCs degeneration without VF defect (23). These inconsistencies in structure and function may also lead to the weak structure-function correlation in those intracranial tumor patients.

In the contrast to the intracranial tumor, in our study, at the same age range and VF MD levels, glaucoma patients have more severe RNFL and optic nerve head damage. The logistic regression analysis shows the RNFL loss tending to the diagnosis of glaucoma; the irregular VF damage is inclined to the diagnosis of intracranial tumor. Few studies had ever explored the RNFL difference between those two VF-affected diseases. This may provide some information for the differential diagnosis.

There are some limitations to our study. First, the smaller number of participants may introduce some selection bias. Second, there are not enough post-geniculate participants to analyze the direct and transsynaptic retrograde degeneration respectively. The same problem exists in the angle-closed and open glaucoma cases. Further study should be conducted to explore more detailed information.

In conclusion, due to few correlation coefficients, intracranial tumor has a weak correlation between the RNFL thickness and Octopus visual field MD, compared with glaucoma. RNFL and optic nerve head damage were more prominent in glaucoma patients when compared to intracranial tumor patients. OCT and Octopus visual field may provide more information for the differential diagnosis of intracranial tumor and glaucoma.

## Data availability statement

The original contributions presented in the study are included in the article. Further inquiries can be directed to the corresponding author.

## Ethics statement

The studies involving human participants were reviewed and approved by Peking University People’s Hospital and Peking University International Hospital. The patients/participants provided their written informed consent to participate in this study.

## Author contributions

XL, JQ, and XC wrote the main manuscript text, and prepared figures and tables. ZR, TC, and YB provided the data. All authors reviewed the manuscript. All authors contributed to the article and approved the submitted version.

## Funding

This study was supported by the National Natural Science Foundation of China (NSFC, No. 21173012) and National Key R&D Program of China, No.2020YFC2008200.

## Conflict of interest

The authors declare that the research was conducted in the absence of any commercial or financial relationships that could be construed as a potential conflict of interest.

## Publisher’s note

All claims expressed in this article are solely those of the authors and do not necessarily represent those of their affiliated organizations, or those of the publisher, the editors and the reviewers. Any product that may be evaluated in this article, or claim that may be made by its manufacturer, is not guaranteed or endorsed by the publisher.
